# Propagated but Topologically Distributed Forebrain Neurons Expressing Alpha-Synuclein in Aged Macaques

**DOI:** 10.1371/journal.pone.0166861

**Published:** 2016-11-18

**Authors:** Katsuo Kimura, Ken-ichi Inoue, Yoshiyuki Kuroiwa, Fumiaki Tanaka, Masahiko Takada

**Affiliations:** 1 Systems Neuroscience Section, Primate Research Institute, Kyoto University, Inuyama, Aichi, Japan; 2 Department of Neurology and Stroke Medicine, Graduate School of Medicine, Yokohama City University, Yokohama, Japan; 3 Department of Neurology and Stroke Center, University Hospital Mizonokuchi, School of Medicine, Teikyo University, Kawasaki, Japan; Northeastern Ohio Medical University, UNITED STATES

## Abstract

In neurodegenerative disorders, such as Parkinson's disease (PD), alpha-synuclein (α-syn) accumulates to induce cell death and/or form a cytoplasmic inclusion called Lewy body (LB). This α-syn-related pathology is termed synucleinopathy. It remains unclear how α-syn accumulation expands during the progress of synucleinopathy in the human brain. In our study, we investigated the patterns of distribution and propagation of forebrain neurons expressing α-syn in aged macaques. It was found that the occurrence of α-syn-positive neurons proceeded topologically based on the midbrain dopamine pathways arising from the substantia nigra and the ventral tegmental area where they were primarily observed. In the nigrostriatal or mesolimbic dopamine pathway, the age-dependent increase in α-syn-positive neurons was evident in the striatum or the nucleus accumbens, respectively. Concerning the nigrostriatal pathway, a mediolateral or rostrocaudal gradient was seen in the substantia nigra or the striatum, respectively, and a compensatory increase in dopamine transporter occurred in the striatum regardless of the decreased dopamine level. In the mesocortical dopamine pathway, α-syn-positive neurons appeared in the prefrontal and then motor areas of the frontal lobe. Given that neither LB formation nor clinical phenotype manifestation was detected in any of the monkeys examined in the present study, aged macaques may be useful as a potential presymptomatic model for PD and LB-related neuropsychiatric disorders.

## Introduction

Alpha-synuclein (α-syn) is a 140-amino acid protein that is localized specifically to the presynaptic terminals and nuclei of neuronal cells, though its physiological functions remain to be known [1,2]. In at least part of neurodegenerative disorders, the onset of cell death is characterized by unusual accumulation of α-syn within the cytoplasm. This α-syn-related pathological condition is generally termed synucleinopathy [3]. The most representative disorder in synucleinopathy is Parkinson’s disease (PD) that is caused by degeneration of dopaminergic neurons in the substantia nigra, resulting in motor impairments due to dysfunction of the nigrostriatal dopamine system [4]. In PD, dopaminergic neuron degeneration is triggered by excess accumulation of phosphorylated α-syn, which leads to the formation of a cytoplasmic inclusion, so-called Lewy body (LB) [5,6]. Thus, LB is a major pathological hallmark characteristic of PD. The α-syn pathology was first defined in familial types of PD [7,8]. For example, type 1 familial PD (PARK1) is ascribed to mutation of the SNCA gene that encodes α-syn [9]. The SNCA gene abnormality results in overexpression and drastic aggregation of α-syn. A similar mechanism underlies type 4 familial PD (PARK4) in which the SNCA gene is triplet and α-syn production is more rapid than in PARK1 [10,11]. It is well known that the α-syn pathology is involved not only in familial types, but also in sporadic cases of PD [12].

Clinical reports have shown that dementia is developed or accompanied in many of PD patients to induce Parkinson’s disease with dementia (PDD) or dementia with Lewy bodies (DLB), respectively [13,14]. Like PD, the α-syn pathology provides a conspicuous sign for these types of dementia [15]. Together with PD, PDD and DLB are classified as Lewy body diseases, and LB is found not only in the substantia nigra, but also extensively in cortical and subcortical regions [16]. Furthermore, PD has various non-motor symptoms, such as psychiatric disorders, gastrointestinal problems, and autonomic failures. These non-motor symptoms appear 10–30 years prior to the onset of motor symptoms. Such a “presymptomatic” state is not fully investigated and is sometimes called incidental LB disease [12].

To understand the pathophysiology of synucleinopathy, it is important to know how α-syn accumulation expands in the process of normal aging. However, the following problems make it very difficult to analyze this issue using human brains. First, the human brain is too huge to undergo pathological examinations throughout the brain. Second, it is quite painful to collect brain samples at individual ages with no history of neuropsychiatric disorders. Instead of human brains, the application of nonhuman primate brains is meritorious for exploring the extent of α-syn accumulation during normal aging. In fact, Chu et al. [17] performed counts of α-syn-positive neurons in the substantia nigra in aged human and monkey brains, and obtained the data showing that the number of α-syn-positive nigral neurons is increased at a similar rate in both of the aged brains. Recently, several studies suggest that the α-syn pathology is transmitted from neuron to neuron in relation to disease development [18–23]. In PD and related disorders, the neuron-to-neuron transmission is most likely to depend on the midbrain dopamine systems, including the nigrostriatal pathway.

In the present study, we investigated the patterns of distribution and propagation of α-syn-positive neurons in aged monkey brains, in order to elucidate when and where synucleinopathy starts and how synucleinopathy develops. Particular attention was paid to forebrain structures, including the frontal cortical areas. In addition, closer analysis was carried out to clarify the age-dependent progress in the α-syn pathology of the nigrostriatal dopamine system. For this work, we used a total of 14 macaque monkeys without abnormal movement sign or past disease history. Their ages ranged 10–31 years old. According to previous reports [24,25], the age of macaques is approximately three times as much as that of humans. Thus, the ages of the monkeys used here are equivalent to 30–93 years old in humans.

## Materials and Methods

The experimental protocol used for this study including animal sacrifice was approved by the Animal Welfare and Animal Care Committee of the Primate Research Institute, Kyoto University (Permission Number: 2013–008). All experiments were conducted in accordance with the Guidelines for Care and Use of Nonhuman Primates (Ver. 3, 2010) issued by the institute.

### Animals

Fourteen macaque monkeys [seven Japanese monkeys (*Macaca fuscata*), six cynomolgus monkeys (*Macaca fascicularis*), and one rhesus monkey (*Macaca mulatta*)] of either sex weighing 4.0–8.5 kg were used for this study. As described in Table 1, the ages of these monkeys were between 10 and 31 years old, corresponding to 30–93 years old in humans [24,25]. All monkeys were supplied from colonies in the Primate Research Institute. The monkeys were originally housed in group cages, and a few days before sacrifice, they were moved to individual cages (780 mm wide x 790 mm deep x 870 mm high; wooded toys provided as environmental enrichment) for precise behavioral inspection. The individual cages were administered on a 12-h on/12-h off lighting schedule and had ad libitum access to food and water. Each of the monkeys had his/her own health record with the birth date; there was no history of either disease or medication. At the time of sacrifice, none of them exhibited any discernible signs of motor impairments.

**Table 1:** List of macaques used. Cynomolgus, cynomolgus monkey (*Macaca fascicularis*); F, female; Japanese, Japanese monkey (*Macaca fuscata*); M, male; m, months; Rhesus, rhesus monkey (*Macaca mulatta*); y, years.

Name	Spieces	Age	Gender
IB	Cynomolgus	10y0m	F
WS	Japanese	14y3m	F
KL	Japanese	19y1m	F
SK	Cynomolgus	20y6m	F
OM	Cynomolgus	21y10m	M
RJ	Japanese	22y0m	F
HU	Cynomolgus	23y2m	M
AY	Cynomolgus	24y0m	F
DD	Japanese	25y11m	F
GF	Cynomolgus	26y7m	M
QR	Japanese	27y0m	F
TK	Japanese	29y4m	F
EK	Rhesus	30y4m	F
DL	Japanese	31y7m	F

### Immunohistochemical procedures

Following sedation with ketamine hydrochloride (10 mg/kg, i.m.) and xylazine hydrochloride (1–2 mg/kg, i.m.), the monkeys were deeply anesthetized with an overdose of sodium pentobarbital (50 mg/kg, i.v.) for perfusion-fixation. The monkeys were transcardially perfused with 0.1 M phosphate-buffered saline (PBS; pH 7.4), followed by 10% formalin in 0.1 M phosphate buffer (PB; pH 7.4). The brains were removed from the skull, postfixed in the same fresh fixative overnight, and saturated with 30% sucrose in PB at 4°C. Frontal sections were then cut serially at 50 μm thickness on a freezing microtome and processed for immunohistochemical staining with the avidin-biotin peroxidase complex (ABC) method. A series of every ten sections were immunostained for α-syn, phosphorylated α-syn, tyrosine hydroxylase (TH), or dopamine transporter (DAT). Especially for TH and DAT immunostaining, the sections through the striatum and the substantia nigra were used. After washes in PBS, the sections were soaked in 0.3% H_2_O_2_ for 30 min to inactivate endogenous peroxidase and immersed in 1% skim milk for 1 h at room temperature. For single bright-field staining, they were incubated at 4°C with mouse monoclonal antibody against α-syn (BD Biosciences, San Jose, CA, USA) at a 1:2,000 dilution, mouse monoclonal antibody against phosphorylated α-syn (Wako, Tokyo, Japan) at a 1:5,000 dilution, rabbit polyclonal antibody against TH (Millipore, Billerica, MA, USA) at a 1:1,000 dilution, or rat monoclonal antibody against DAT (Millipore) at a 1:1,000 dilution, in PBS containing 0.1% Triton X-100 and 2% normal donkey serum. After 48 h, the sections were washed in PBS and incubated in the same fresh medium containing biotinylated goat anti-mouse/anti-rabbit/anti-rat IgG antibody (Vector Laboratories, Burlingame, CA, USA; diluted at 1:200) for 2 h at room temperature, followed by avidin-biotin-peroxidase complex (ABC Elite, Vector Laboratories) for 2 h at room temperature. Subsequently, the sections were reacted in in 0.05 M Tris-HCl buffer (pH 7.6) containing 0.04% diaminobenzidine tetrahydrochloride (Merck, Darmstadt, Germany), 0.04% NiCl_2_, and 0.002% H_2_O_2_. Finally, the sections were mounted onto gelatin-coated glass slides, air-dried, counterstained with 1% Neutral red, and coverslipped. Another series of sections was Nissl-stained with 1% Cresyl violet.

For double immunofluorescence staining for α-syn and TH or TH and NeuN, the sections through the substantia nigra were incubated with a cocktail of the mouse anti-α-syn antibody (BD Biosciences; diluted at 1:1,000) and the rabbit anti-TH antibody (Millipore; diluted at 1:1,000), or a cocktail of the rabbit anti-TH antibody (Millipore; diluted at 1:1,000) and mouse monoclonal antibody against NeuN (Millipore; diluted at 1:2,000). The sections were then incubated with a cocktail of Alexa Fluor 555-conjugated donkey anti-mouse IgG antibody (Molecular Probes, Eugene, OR, USA; diluted at 1:300) and Alexa Fluor 488-conjugated donkey anti-rabbit IgG antibody (Molecular Probes; diluted at 1:200). Images of sections were digitally captured at x4 or x10 magnification using an optical microscope equipped with a high-grade charge-coupled device (CCD) camera (Biorevo, Keyence, Osaka, Japan).

### Quantitative analyses

Anatomical structures were identified by referring to “A Combined MRI and Histology Atlas of the Rhesus Monkey Brain in Stereotaxic Coordinates” [26] and “The Rhesus Monkey Brain in Stereotaxic Coordinates” [27].

In individual monkeys, the numbers of α-syn-positive and TH-positive neurons were counted in the ventral midbrain, including the substantia nigra pars compacta (SNc) and the ventral tegmental area (VTA). Cell counts were performed in every ten sections 50 μm thick (a total of 8–10 sections per monkey) on each side, and the data obtained were averaged and expressed as the number in the area of 1 mm^2^. The entire process was done on digitally captured x40 images by the aid of Adobe Photoshop CS5 software and Adobe Illustrator CS5 software. When the numbers of α-syn-positive and TH-positive neurons were compared in the medial vs. lateral parts of the SNc, 3 regions of interest (ROIs) each of which was 200 μm x 200 μm were placed in both parts along the mediolateral axis. In each part, the cell counts obtained in the ROIs were averaged and converted into the number in the area of 1 mm^2^. The same process as described above was taken for analysis.

The number of α-syn-positive neurons was also counted in forebrain structures, including the frontal cortical areas. In each structure, cell counts were performed in every ten sections 50 μm thick (a total of 2–6 sections per monkey) on each side. On each section, 3 ROIs of 200 μm x 200 μm were placed. The data obtained in the ROIs were averaged, and then the neuron number per section was calculated.

For double immunofluorescence staining for α-syn and TH or TH and NeuN, cell counts were carried out in 3 ROIs of 200 μm x 200 μm that were placed within nigral zones where clusters of TH-positive neurons were located in every ten sections (a total of 8–10 sections per monkey).

In individual monkeys, the density of TH immunoreactivity or DAT immunoreactivity was measured in the striatum. Measurements of optical density (OD) were performed in 2 ROIs of 500 μm x 500 μm placed in each of the caudate nucleus and the putamen in every ten sections (a total of 12–16 sections per monkey) on each side. The OD was measured by the aid of Adobe Photoshop CS5 software and Adobe Illustrator CS5 software, and the value obtained in the ROIs in each section was averaged and expressed as a ratio to a control value (OD taken from the anterior limb of the internal capsule). The entire process was done on digitally captured x40 images by the aid of Adobe Photoshop CS5 software. Finally, the mean OD ratio per section was calculated. When the OD ratio was compared in the rostral vs. caudal levels of the striatum, the boundary was set at the level of the anterior commissure.

The cell counts and OD ratios were analyzed by Mann-Whitney test. Correlations between the cell counts and the age, or those between the OD ratios and the age were examined. Significance for each analysis was set a priori at 0.05. All the statistical analyses were done by the aid of IBM SPSS Statistics 22 package and Microsoft Excel 2010.

## Results

As listed in Table 1, 14 macaque monkeys (seven Japanese monkeys, six cynomolgus monkeys, and one rhesus monkey) ranging 10–31 years old were used for the present analyses. These monkeys had no history of diseases or medications. All the monkeys were basically in healthy conditions, and virtually no motor abnormalities were observed in any individuals.

### Age-dependent changes in the distribution of α-syn-positive neurons in the nigrostriatal dopamine system

**Fig 1:**
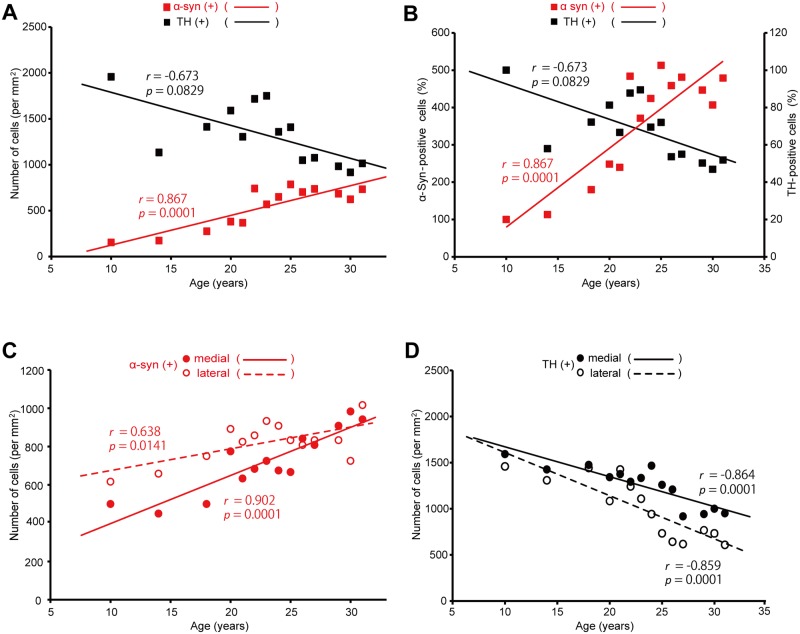
Age-dependent distribution changes in α-syn-positive and TH-positive neurons in the SNc. A: Number of α-syn-positive or TH-positive neurons in the area of 1 mm^2^. The red or black line with filled squares represents the α-syn-positive or TH-positive neuron number, respectively. B: Ratio of the α-syn-positive (specified by the red line with filled squares) or TH-positive (specified by the black line with filled squares) neuron number to each number at 10 years of age. C: Number of α-syn-positive neurons in the medial or lateral part of the SNc. The neuron number is indicated as that in the area of 1 mm^2^. The solid line with filled circles or broken line with open circles represents the neuron number in the medial or lateral part, respectively. D: Number of TH-positive neurons in the medial (denoted by the solid line with filled circles) or lateral (denoted by the broken line with open circles) part of the SNc. The neuron number is indicated as that in the area of 1 mm^2^. See the text for methodological details.

**Fig 2:**
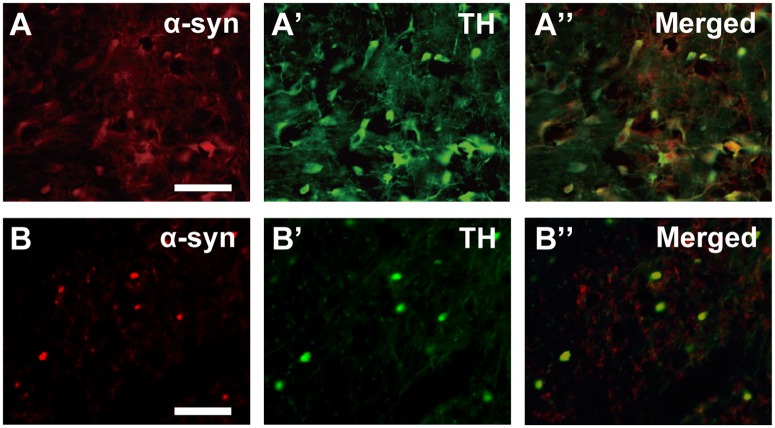
Photomicrographs of double immunofluorescence staining for α-syn and TH in SNc at 10 (A,A’,A”) and 31 (B,B’,B”) years of age. Note that the intensity of α-syn is weak in many TH-positive neurons at 10 years of age, and that the vast majority of TH-positive neurons are clearly immunostained for α-syn at 31 years of age. Scale bars, 50 μm.

**Fig 3:**
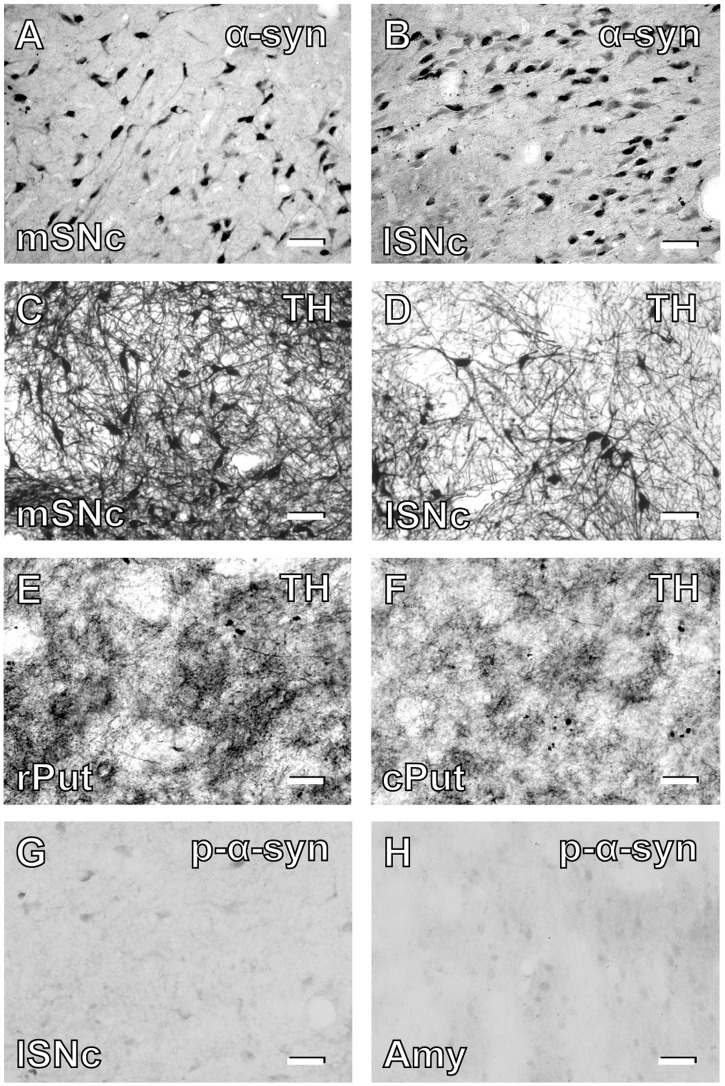
Photomicrographs of α-syn and TH immunostaining in the nigrostriatal dopamine system and forebrain structures at 31 years of age. A: α-Syn-positive neurons in the medial part of the SNc (mSNc). B: α-Syn-positive neurons in the lateral part of the SNc (lSNc). C: TH-positive neurons in the mSNc. D: TH-positive neurons in the lSNc. E: TH-positive fibers and terminals in the rostral aspect of the putamen (rPut). F: TH-positive fibers and terminals in the caudal aspect of the putamen (cPut). G: Phosphorylated α-Syn immunostaining in the lSNc. G: Phosphorylated α-Syn immunostaining in the amygdala (Amy). Scale bars, 50 μm.

In the youngest 10-year-old monkey, α-syn-positive neurons were found prominently in the SNc (Table 2). Therefore, we first analyzed the age-dependent changes in the distribution of α-syn-positive neurons in the nigrostriatal dopamine system. At 10 years of age, a number of α-syn-positive neurons were already located in the SNc, though α-syn immunoreactivity in each neuron was not so intense as compared to that at older ages. As the age went, α-syn-positive neurons in the SNc gradually increased in both number and intensity (Figs 1A and 2). By 30 years of age, the number of α-syn-positive neurons was increased about five times as many as that at 10 years of age (Fig 1B). Conversely, the number of TH-immunoreactive neurons in the SNc was decreased by more than 50% with the age (Fig 1A and 1B). Double immunofluorescence histochemistry confirmed that substantially all α-syn-positive neurons were immunoreactive for TH throughout all the ages examined (Fig 2). These overall results suggest that nigral dopamine neurons in aged macaque brains come to express α-syn frequently, and that at least some of them degenerate to death. Around 30 years of age, the number of α-syn-positive neurons became close to that of TH-immunoreactive neurons, indicating that most of the surviving nigral dopamine neurons express α-syn definitively. We further examined the possible difference in α-syn-positive neuron distribution in the mediolateral direction of the SNc. At younger ages, α-syn-positive neurons were distributed more frequently in the lateral than in the medial part of the SNc (*p* = 0.0362, Figs 1C, 3A and 3B). The α-syn-positive neuron numbers in the medial vs lateral parts became close or almost the same or even reversed at older ages than 25 years (Fig 1C). By contrast, more TH-immunoreactive neurons were located in the medial than in the lateral part of the SNc at substantially all ages (*p* = 0.0278, Figs 1D, 3C and 3D). In none of the monkeys used here, abnormal inclusions reminiscent of LB were detected in nigral dopamine neurons.

**Table 2:** Distribution patterns of α-syn-positive neurons in subcortical structures. -, none; +, sparse (1-10/ROI); ++, moderate (11-20/ROI); +++, dense (21-/ROI). Amy, amygdala; Hip, hippocampus; LS, lateral septum; m, months; NA, nucleus accumbens; RRF, retrorubral field; SNc, substantia nigra pars compacta; Str, striatum; VTA, ventral tegmental area; y, years.

Name	Age	SNc	VTA	RRF	Str	NA	LS	Amy	Hip
IB	10y0m	++	+	+	-	-	-	+	+
WS	14y3m	++	+	+	-	-	-	+	++
KL	19y1m	++	+	+	-	-	-	+	++
SK	20y6m	+++	+	+	+	-	-	+	++
OM	21y10m	+++	++	+	-	+	-	+	++
RJ	22y0m	+++	++	+	+	-	-	+	++
HU	23y2m	+++	++	++	+	+	-	+	++
AY	24y0m	+++	++	+	+	+	+	+	++
DD	25y11m	+++	++	++	+	+	+	+	+++
GF	26y7m	+++	++	+	+	+	+	++	+++
QR	27y0m	+++	++	++	++	+	+	++	+++
TK	29y4m	+++	++	+	++	+	+	+++	+++
EK	30y4m	+++	+++	++	++	++	+	++	+++
DL	31y7m	+++	+++	++	++	++	+	+++	+++

**Fig 4:**
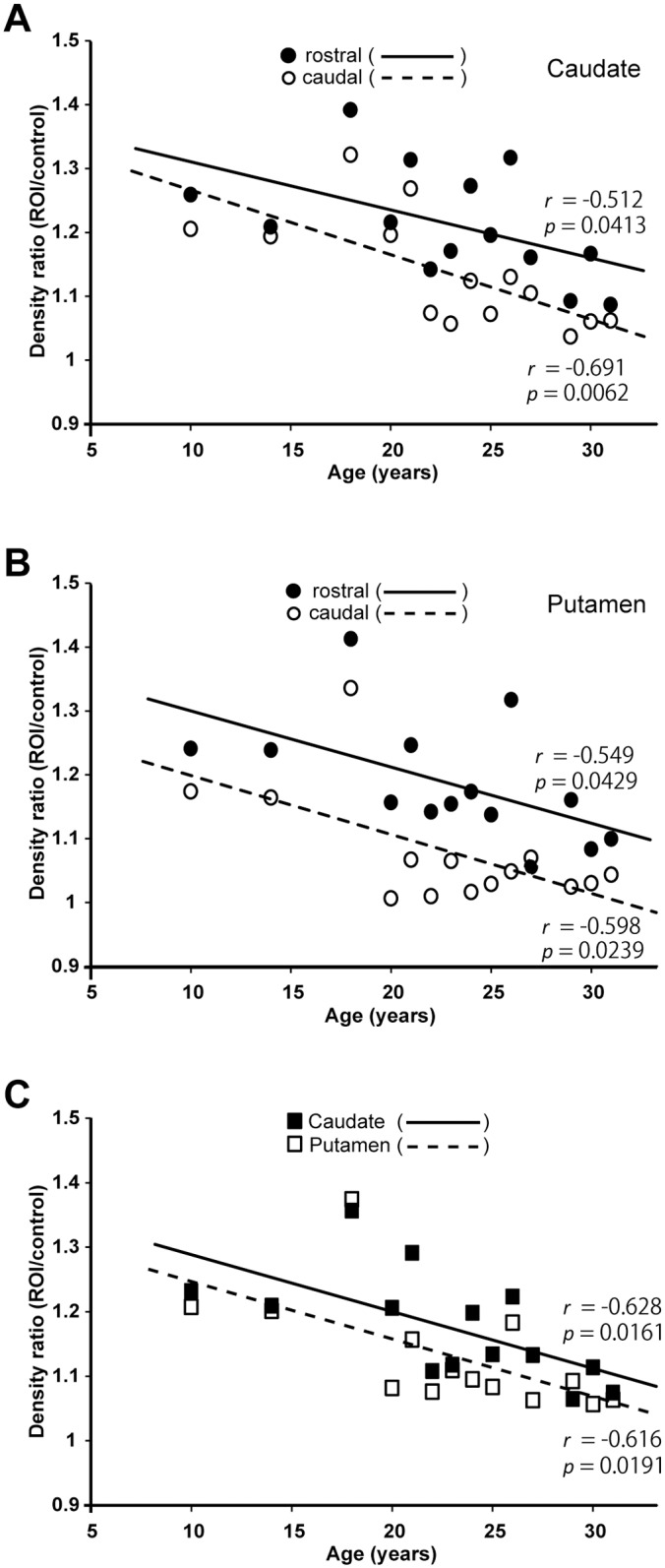
Age-dependent density changes in TH immunoreactivity in the striatum. A: Change in TH immunoreactivity in the caudate nucleus at the rostral or caudal level. The density ratio was measured in comparison with background staining in the internal capsule as a control. The solid line with filled circles or broken line with open circles represents the density ratio at the rostral or caudal level, respectively. B: Change in TH immunoreactivity in the putamen at the rostral (denoted by the solid line with filled circles) or caudal (denoted by the broken line with open circles) level. C: Change in TH immunoreactivity in the caudate nucleus (specified by the solid line with filled circles) or the putamen (specified by the broken line with open circles). See the text for methodological details.

**Fig 5:**
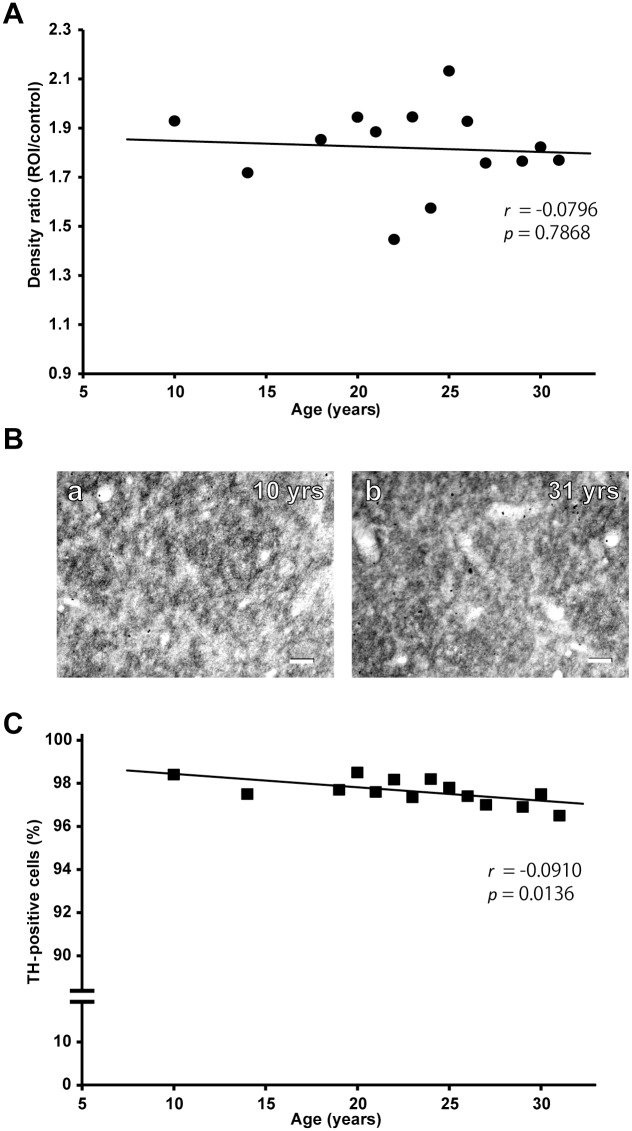
Age-dependent changes in striatal DAT immunoreactivity and nigral TH-positive neurons. A: Age-dependent change in DAT immunoreactivity in the striatum. The density ratio was measured in comparison with background staining in the internal capsule as a control. B: Photomicrographs of DAT immunostaining in the striatum at 10 (a) and 31 (b) years (yrs) of age. Scale bars, 50 μm. C: Age-dependent change in the ratio of TH-positive neurons to NeuN-positive neurons in the SNc. Data are obtained from double immunofluorescence staining for TH and NeuN. See the text for methodological details.

The density of TH-immunoreactive fibers and terminals in the striatum was gradually reduced as the age went (Fig 4A for the caudate nucleus and Fig 4B for the putamen). Throughout all the ages tested, TH immunoreactivity was seen less intensely in the caudal than in the rostral aspect of the striatum (caudate; *p* = 0.0276, putamen; *p* = 0.0045, Figs 3E, 3F, 4A and 4B). Moreover, comparison between the caudate nucleus and the putamen revealed that TH immunoreactivity was less densely observed in the putamen than in the caudate nucleus regardless of the age (*p* = 0.0354, Fig 4C). When DAT immunoreactivity that was localized at the presynaptic terminals was also examined in the striatum, it was found that unlike the TH-immunoreactive terminal density, the density of DAT-immunoreactive terminals was not essentially reduced even in the far-aged brains (Fig 5A and 5B). Moreover, double immunofluorescence histochemistry for TH and NeuN confirmed that the vast majority of NeuN-positive neurons in the SNc displayed TH immunoreactivity throughout all the ages examined (Fig 5C). This implies that even at older ages, most of the surviving neurons in the SNc are dopaminergic (i.e., they retain the capability of dopamine synthesis), because they still express TH.

Although α-syn-positive fibers and terminals in the striatum seemed incremental with the age, their density could not to be analyzed quantitatively in an exact manner. More interestingly, α-syn-positive neurons appeared in the striatum at older ages than 20 years and gradually increased in number (Table 2).

### Distribution and propagation of α-syn-positive neurons in subcortical structures

In monkeys younger than 20 years old, α-syn-positive neurons were located not only in the SNc, but also in the VTA, the retrorubral field, the amygdala, and the hippocampus (Table 2). As the age went, the numbers of α-syn-positive neurons in these subcortical structures were gradually increased, and reached a similar extent to that in the SNc between 25 and 30 years of age (Table 2). As described above, a number of α-syn-positive neurons were observed in the striatum at older ages than 20 years (Table 2), and, in addition, α-syn-positive neurons emerged in the nucleus accumbens and the lateral septum by 25 years of age (Table 2). Especially in the striatum and the nucleus accumbens, the numbers of α-syn-positive neurons were kept increased constantly until over 30 years of age (Table 2). The distribution of α-syn-positive neurons within the striatum exhibited a rostrocaudal gradient; they were located more frequently in the caudal than in the rostral aspect of the striatum. This topological pattern was seen more prominently at older ages (data not shown). Throughout all the ages tested, unusual inclusions like LB were not found in any subcortical structures.

### Distribution and propagation of α-syn-positive neurons in frontal cortical areas

**Fig 6:**
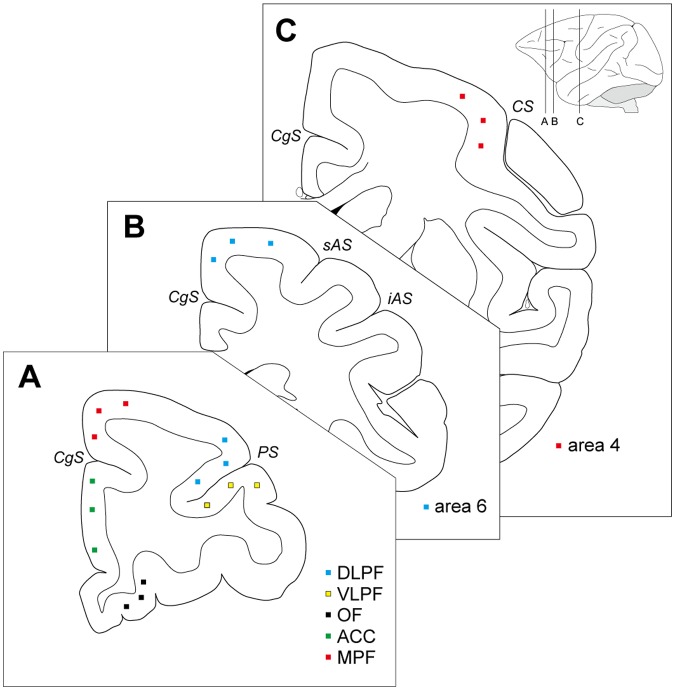
Schematic drawings of the approximate locations of the frontal cortical areas for analyzing the density of α-syn-positive neurons. Three representative frontal sections are arranged rostrocaudally in A-C. The rostrocaudal levels of the sections are indicated at the top-right corner. Modified from “The Rhesus Monkey Brain in Stereotaxic Coordinates” [27]. ACC, anterior cingulate cortex; CgS, cingulate sulcus; CS, central sulcus; DLPF, dorsolateral prefrontal cortex; iAS, inferior limb of the arcuate sulcus; MPF, medial prefrontal cortex; OF, orbitofrontal cortex; PS, principal sulcus; sAS, superior limb of the arcuate sulcus; VLPF, ventrolateral prefrontal cortex.

Throughout all the ages examined, both the anterior cingulate cortex and the orbitofrontal cortex contained α-syn-positive neurons (Table 3; Fig 6A). The numbers of α-syn-positive neurons in these frontal cortical areas were markedly increased with the age (Table 3). Furthermore, around 20 years of age, α-syn-positive neurons were located in the prefrontal cortex, especially in the dorsolateral prefrontal cortex, and in area 6 of the motor cortex (i.e., the premotor cortex and the supplementary motor area) (Table 3; Fig 6A and 6B). The numbers of α-syn-positive neurons in these areas were gradually increased as the age went (Table 3). At much older ages, α-syn-positive neurons appeared in other areas of the prefrontal cortex, such as the ventrolateral and medial prefrontal cortices, and in area 4 of the motor cortex (i.e., the primary motor cortex) (Table 3; Fig 6A and 6C). In none of the monkeys used here, abnormal inclusions like LB were seen in any frontal cortical areas.

**Table 3:** Distribution patterns of α-syn-positive neurons in frontal cortical areas. -, none; +, sparse (1-10/ROI); ++, moderate (11-20/ROI); +++, dense (21-/ROI). ACC, auterior cingulate cortex; Area 4, area 4 of the motor cortex; Area 6, area 6 of the motor cortex; DLPF, dorsolateral prefrontal cortex; MPF, medial prefrontal cortex; m, months; OF, orbitofrontal cortex; VLPF, ventrolateral prefrontal cortex; y, years.

Name	Age	ACC	OF	DLPF	VLPF	MPF	Area 6	Area 4
IB	10y0m	+	+	-	-	-	-	-
WS	14y3m	+	+	-	-	-	-	-
KL	19y1m	+	+	+	-	-	+	-
SK	20y6m	+	+	-	-	-	-	-
OM	21y10m	++	++	+	-	-	+	-
RJ	22y0m	++	++	-	-	-	+	-
HU	23y2m	++	++	+	+	+	+	-
AY	24y0m	++	++	+	+	-	+	+
DD	25y11m	++	++	+	-	-	+	-
GF	26y7m	+++	+++	++	+	+	+	-
QR	27y0m	++	+++	++	+	-	+	-
TK	29y4m	+++	+++	++	+	+	+	+
EK	30y4m	+++	+++	++	+	+	+	+
DL	31y7m	+++	+++	++	+	+	++	+

### Occurrence of phosphorylated α-syn neurons

Using three representative monkeys at the ages of 20–30 years (monkeys SK, DD, and EK), we examined whether or not phosphorylated α-syn-positive neurons occur in the aged brain. In all of these monkeys, only a few faintly-immunostained neurons were observed in the SNc and the amygdala (Fig 3G and 3H). No other structures contained very few, if any, phosphorylated α-syn-positive neurons.

## Discussion

In present study, we analyzed the patterns of distribution and propagation of forebrain neurons expressing α-syn in aged macaques. The main findings were as follows: (1) α-Syn-positive neurons appeared topologically along the midbrain dopamine pathways originating in the substantia nigra and the ventral tegmental area where they were primarily located. (2) Regarding the nigrostriatal or mesolimbic dopamine pathway, α-syn-positive neurons were increasingly observed in the striatum or the nucleus accumbens, respectively, in an age-dependent fashion. (3) In the nigrostriatal pathway, a mediolateral or rostrocaudal gradient was seen in the substantia nigra or the striatum, respectively. (4) Concerning the mesocortical dopamine pathway, α-syn-positive neurons were found in the prefrontal and then motor areas of the frontal lobe.

It is generally accepted that in neuropathological conditions, phosphorylated α-syn abnormally accumulates within the cytoplasm and aggregates to form insoluble fibrils characterized by the intracellular inclusion LB [5]. This α-syn-related event termed synucleinopathy is widely involved not only in PD, PDD, and DLB, but also in multiple system atrophy and part of Alzheimer’s disease (AD) [7,8,28,29]. In order to understand the progress in synucleinopathy, it is essential to study how α-syn-positive neurons propagate during normal aging by using nonhuman primates. A previous report showed that α-syn-positive neurons were found in the substantia nigra, the striatum, and the cerebral cortex in the aged lemur primate brain [30]. However, it still remains unknown exactly how the α-syn pathology develops. To elucidate when and where synucleinopathy begins and how synucleinopathy proceeds, we explored the patterns of distribution and propagation of α-syn-positive neurons in aged monkey brains. In the present study, 14 macaques (seven Japanese monkeys, six cynomolgus monkeys, and one rhesus monkey) ranging 10–31 years old were used. Based on the fact that the age of macaques is about three times as much as that of humans [24,25], the ages of these monkeys correspond to 30–93 years old in humans. All data obtained in individual monkeys were dealt with together across the species, because there was no marked species difference in the age-dependent progress in the α-syn pathology.

According to the hypothesis proposed by Braak [31], in PD patients, LB formation first occurs in the brainstem and the olfactory bulb, and gradually spreads to the midbrain, the basal ganglia, and the cerebral cortex. This process of extension of LB formation may not necessarily be disease-specific, but may commonly be seen during normal aging as well. Here we investigated the distribution of α-syn-positive neurons in the forebrain, including cortical and subcortical regions, of aged macaques. As previously reported [17], the number of α-syn-positive neurons in the SNc is increased with the age. Conversely, the number of dopaminergic (TH-positive) neurons is decreased in the SNc. The older the age becomes, the more frequently nigral dopamine neurons express α-syn. By 30 years of age, the α-syn-positive neurons reach five times more than those at 10 years of age, and, finally, most of the remaining nigral dopamine neurons express α-syn. It is known that familial PD, i.e., PARK1 and PARK4, is characterized by rapid α-syn accumulation and early disease onset [32]. On the other hand, the disease onset is usually seen late (at older ages) in most cases of sporadic PD in which as many as 60–80% of nigral dopamine neurons are damaged when motor symptoms occur [33–36]. In this context, it is quite reasonable that none of the monkeys used in the present study exhibited definite signs for PD, because nearly 50% of nigral dopamine neurons are still retained even in the far-aged monkeys. This implies that the brains in these monkeys may be in presymptomatic conditions of PD. In addition, it should also be emphasized here that substantially no phosphorylated α-syn-positive neurons appeared in any aged monkey brain.

It has been shown that there is topological arrangement in the nigrostriatal dopamine pathway [37–39]. Neurons in the medial part of the SNc project predominantly to the rostral aspect of the striatum (especially to the caudate nucleus and the rostral putamen; striatal associative territory), while those in the lateral counterpart project mainly to the caudal aspect of the striatum (especially to the caudal putamen; striatal motor territory). Recent emphasis has been placed on the functional heterogeneity as to reward-related response; the former neurons encode motivational values, and the latter neurons encode motivational salience [40]. The present results have demonstrated that throughout all ages, the dopaminergic neurons in the lateral SNc are affected more severely than those in the medial SNc. In favor of these findings, it has been reported in human PD cases that the pathological change in the SNc is the most prominently observed in the ventrolateral tier [41–44]. The age-related distribution pattern of α-syn-positive neurons in the SNc is reversed. The α-syn-positive neurons are located more numerously in the lateral SNc than in the medial SNc, especially at the earlier stage of aging. In accordance with the topological organization of the nigrostriatal dopamine pathway, loss of dopaminergic fibers and terminals from the striatum occurs more markedly at its caudal level (particularly in the caudal putamen) receiving input from the lateral SNc, than at its rostral level receiving input from the medial SNc. A previous work [44] has described that the density of dopaminergic fibers and terminals is reduced in the putamen by 35–75% one-to-three years after motor symptoms appeared in human PD cases. Although the actual extent of decreased striatal TH immunoreactivity was not necessarily measured in our study, an insufficient reduction of striatal dopamine for developing parkinsonian signs is most likely in the aged monkeys examined here, thus suggesting that they may provide a presymptomatic model for PD.

Contrary to the decreased density of TH immunoreactivity, DAT immunoreactivity in the striatum remains unchanged even in the far-aged brains. Striatal DAT is localized at the presynaptic terminals of nigral dopamine neurons to promote reuptake of dopamine that is released from the terminals [45]. This clearly indicates that the striatal DAT immunoreactivity represents the dopaminergic axon terminals. To explain a serious discrepancy between the density of TH and DAT immunoreactivity, it is likely to consider that DAT may at least in part be placed at terminal sites of damaged or degenerating nigrostriatal neurons that can no longer synthesize dopamine. However, our double immunofluorescence histochemistry for TH and NeuN has revealed that even at older ages, the surviving neurons in the SNc apparently express dopamine and thus do not lose the capability of dopamine synthesis. The discrepancy could be accounted for by postulating that DAT expressed in the remaining dopamine neurons might display hypersensitivity to maintain intracellular dopamine concentrations. In favor of this notion, previous works have reported that DAT binding in the striatum is increased in a compensatory manner at early stages of PD onset in MPTP-treated monkeys [46,47].

It has recently been revealed that the α-syn pathology related to disease development is transmitted based on the neural linkage [18–23]. The present study defines that α-syn-positive neurons propagate topologically in accordance with the midbrain dopamine systems, as evidenced by the findings that α-syn-positive neurons appear in the striatum in an age-dependent fashion based on dopaminergic input loss along the nigrostriatal pathway responsible for PD. The α-syn-positive neurons in the striatum occur later than in the SNc and are located in a topological manner in which they are seen more markedly in the caudal than in the rostral aspect. This rostrocaudal gradient of α-syn-positive neuron distribution within the striatum corresponds well with the topological pattern of loss of dopaminergic fibers and terminals therefrom.

Our results have further demonstrated that the number of α-syn-positive neurons is increased in the VTA as the age goes, though they appear less rapidly and drastically than in the SNc. In conjunction with the medial SNc, the VTA gives rise to the mesolimbic and mesocortical dopamine pathways [48,49]. The mesolimbic dopamine pathway terminates predominantly in the nucleus accumbens. With the age, α-syn-positive neurons emerge in the nucleus accumbens in which they appear later than in the striatum. Part of the mesolimbic dopamine pathway from the VTA/medial SNc projects to the amygdala, the hippocampus, and the lateral septum. In view of the fact that these projections are not so strong as the projection to the nucleus accumbens [50], it does not seem possible that both the amygdala and the hippocampus are major subcortical structures that are subjected to α-syn accumulation from the early stage of aging. This might be explained by considering that the α-syn pathology of these structures is linked not only with the mesolimbic dopamine system, but also probably with the development in dementia-related diseases. With respect to the human brain pathology of dementia-related diseases, especially PDD, DLB, and part of AD, it has been documented that LB formation is observed primarily in the hippocampus, the amygdala, and the temporal lobe [8]. Although the Braak’s hypothesis [31] is generally adopted for pathological progression of PD based on LB formation, only PD cases without dementia are dealt in this hypothesis. The stage classification of PD has been extended with dementia cases included [8]. This describes that the LB pathology originates from the olfactory bulb and spreads to the amygdala and the temporal lobe. Furthermore, Baba et al. [51] have recently reported that PD cases with olfactory dysfunctions often result in atrophy of the amygdala and then in cognitive declines. Taken together, α-syn accumulation in the hippocampus and the amygdala develops the pathology of PDD and DLB. Similarly, the α-syn accumulation therein is exhibited in part of AD, leading to cognitive declines [8].

Moreover, neurons in the VTA/medial SNc provide the mesocortical dopamine pathway to the frontal lobe [3,38,52], and, therefore, a large part of the frontal lobe is thought to receive dopaminergic innervation in varying degree [53–55]. It has been shown in the present study that numbers of α-syn-positive neurons are located in the anterior cingulate cortex and the orbitofrontal cortex from the early stage of aging, and that their age-dependent increment is seen at a similar or even higher rate than in the VTA. This suggests that the α-syn pathology of these frontal cortical areas may be ascribable to other mechanisms (for instance, dementia-related disease progression), as well as to the mesocortical dopamine system. At the later stage of aging, the occurrence of α-syn-positive neurons expands to the prefrontal cortex, in particular abundance in the dorsolateral prefrontal cortex, and in area 6 of the motor cortex (i.e., the premotor cortex and the supplementary motor area). Then, the appearance of α-syn-positive neurons finally reaches other areas of the prefrontal cortex, such as the ventrolateral and medial prefrontal cortices, and in area 4 of the motor cortex (i.e., the primary motor cortex). Although all these frontal cortical areas are likely to receive dopaminergic input from the VTA/medial SNc [53–56], it remains unclear whether the development in the α-syn pathology may be correlated with the richness of dopaminergic innervation. There is a consensus that the prefrontal cortical areas, including the anterior cingulate cortex and the orbitofrontal cortex, are greatly involved in diverse cognitive functions [38,52,57,58]. In this context, the α-syn pathology of the frontal lobe might affect cognitive deficits manifested not only in PD, but also in the dementia-related diseases, such as PDD, DLB, and part of AD.

The present results indicate that the α-syn pathology expands primarily along the dopaminergic neuron connectivity in a topological fashion. At the early stage of aging, α-syn-positive neurons emerge in midbrain dopamine neurons. From the SNc, the occurrence of α-syn-positive neurons proceeds to the striatum through the nigrostriatal dopamine pathway, while from the VTA/medial SNc, it extends to the limbic structures via the mesolimbic dopamine pathway, and to the frontal cortical areas via the mesocortical dopamine pathway. This propagation pattern of α-syn-positive neurons seems basically compatible to the human LB pathology. In none of the aged macaque monkeys tested in the present study, however, either LB formation or clinical manifestation (at least PD-like motor signs) was detected. This indicates that the α-syn pathology is not necessarily disease-specific, but is common to normal aging. It should also be noted here that not only the propagation associated with the midbrain dopamine systems, but also the dementia-related changes probably underlie the α-syn pathology, especially in the amygdala, the hippocampus, the anterior cingulate cortex, and the orbitofrontal cortex. Overall, the aged macaques may be useful as a potential presymptomatic model for PD and, also, for other types neuropsychiatric disorders such as PDD, DLB, and AD.
